# Evaluation of prognostic scoring systems in patients hospitalized from the emergency department in a low-income region: northern Syria after internal turmoil as a different universe

**DOI:** 10.55730/1300-0144.5595

**Published:** 2022-12-13

**Authors:** Burak ÇELİK, Bahadır KARACA

**Affiliations:** 1 Department of Emergency, Kırşehir Training and Research Hospital, Kırşehir, Turkey; 2 Department of Emergency, Sancaktepe Şehit Prof. Dr. İlhan Varank Training and Research Hospital, İstanbul, Turkey

**Keywords:** Emergency department, HOTELS, NEWS2, RAMS, RAPS, Syria

## Abstract

**Background/aim:**

In low-income or underdeveloped countries with conflict and internal unrest, healthcare facilities and staff are limited. For these reasons, it is necessary to use the most straightforward scoring systems to ensure that health facilities and staff are used effectively and to expedite processes through early and effective interventions for patients. In this study, we evaluate and compare the scoring systems used to predict patient prognosis for Emergency Department (ED) patients in northern Syria, which is an area marred by conflict and internal unrest.

**Materials and methods:**

In this study, patients hospitalized in the Afrin, Azez Vatan, Jarablus, Tel Abyad, Rasulayn, El Bab, and Çobanbey hospitals in northern Syria were investigated. Only patients that were hospitalized in the emergency departments of these hospitals, including wards and intensive care units, were included in the study. Patients that were hospitalized from 03/01/2021 to 08/31/2021, the study period, were prospectively analyzed. Vital signs, medical histories and demographic data of the patients were recorded by calculating National Early Warning Score 2 (NEWS2), Rapid Acute Physiology Score (RAPS), Rapid Emergency Medicine Score (REMS), and HOTEL Score (hypotension, oxygen saturation, low temperature, electrocardiogram, loss of independence). Acceptance parameters and scores were analyzed using statistical methods and by comparing groups.

**Results:**

All four scoring systems were found to be effective in predicting mortality regarding ROC curve analysis. However, the statistical significance of the RAPS was slightly stronger than that of the other scores and REMS had the highest sensitivity and specificity amongst the four systems, at 86.2% and 84.1%, respectively. Regarding the risk of hospitalization in the ICU (p < 0.05), the sensitivity values of the cut-off values offered by the scoring systems remained below 0.70 regarding ROC curve analysis. RAPS had the highest sensitivity (65.2%) of the four systems with a cut-off value of 1.5.

**Conclusion:**

This study in northern Syria has shown that although RAPS had stronger statistical power, REMS had better sensitivity and specificity for the prediction of mortality. Additionally, RAPS had better sensitivity for ICU risk. This study will contribute to the evaluation of healthcare in similar regions and to cost-effective healthcare delivery by using scoring systems for ED patients’ admission.

## 1. Introduction

Over the past 30 years, numerous risk classification and scoring systems have been developed for patient populations, such as major trauma [1], critical illness [2], and acute coronary syndrome [3] that can lead to fatal emergencies. Over the past decade, these scoring systems have been diversified, further developed, and recommended for critical patient selection and emergency patient prioritization because of the increasing workload in Emergency Departments (EDs) [4,5].

The purpose of these scoring systems is to calculate risk based on the patient’s physiological or laboratory values. These systems are important in improving the clinical care of patients by providing estimates of the length of hospital stay, length of Intensive Care Unit (ICU) stay, and the mortality risk of patients [4,6].

Emergency medical admissions represent an important part of the healthcare system’s workload [7], and ED length of stay is an important quality indicator of patient care in healthcare facilities [8].

Prognostic predictions are important in identifying critically ill patients in EDs and initiating effective and rapid treatment for these patients [4,9]. For clinical and quality reasons, general risk scoring systems continue to be developed for EDs [8]. An ideal risk score for emergency care should be able to predict clinically serious conditions, such as mortality and the need for ICU admission, based on a limited number of physical or laboratory values that can be quickly and easily obtained [5,10–12]. Currently, parameters such as vital signs, medical history data, and laboratory results are used in different scoring systems [9]. Therefore, scoring systems that can predict mortality across case groups are needed for emergency medical admissions. However, these systems require nonrandomized comparisons of mortality estimates in relevant populations [13].

In developed countries with high-income levels, many scoring systems are used to ensure standardization and high-quality health care. These systems are validated in-country populations or in multicenter studies involving developed countries [14]. In low-income or underdeveloped countries, these studies are a popular subject of research [15]. However, in countries which have conflict and internal unrest have limited healthcare facilities and staff, it is necessary to use the simplest scoring systems to promote the effective use of healthcare facilities and staff. Furthermore, this will expedite processes through early and effective interventions for patients. However, it is challenging to conduct studies to evaluate prognostic scoring systems for populations in these regions.

Syria ranks 5th on the 2022 Global Terrorism Index.^1^ Humanitarian organizations have provided medical care in northern Syria, which has experienced internal unrest and conflict since 2010 [16]. During the conflicts, many healthcare workers were attacked, lost their lives, were arrested, or were forced to emigrate because of harsh conditions [17]. The limited number of remaining healthcare workers had to provide elective and emergency care to more than 4 million people [18].

In this context, decision making processes for patients can be very intense and sudden incapacitation may occur during mass deployments. There have been no studies that have evaluated prognostic scoring systems for patients hospitalized in this, or a similar, regional population to improve clinical assessment and expedite hospitalization and discharge. This study aims to evaluate and compare the scoring systems that are used to predict the prognosis of patients admitted from an ED in northern Syria, where there are limited facilities and healthcare workers following conflict and internal turmoil.

## 2. Materials and methods

### 2.1. Study design

This study investigates the vital signs, medical history data, and demographic data of 1310 patients who were admitted from the emergency departments to the wards and intensive care units between 03/01/2021 and 08/31/2021 in hospitals in northern Syria under the auspices of Turkey. This study is a descriptive study and data were recorded prospectively. Before the start of the study, approval was obtained from the Ethics Committee of Hatay Mustafa Kemal University for noninterventional research (date of meetings: 02/18/2021, number of decisions: 10) and the relevant hospital administrations. Informed consent was obtained from the patients included in the study and their first-degree relatives of the unconscious patients. Additionally, the study was conducted in accordance with the “World Medical Association Declaration of Helsinki Ethical Principles”.

### 2.2. Location of the study

**Figure 1:**
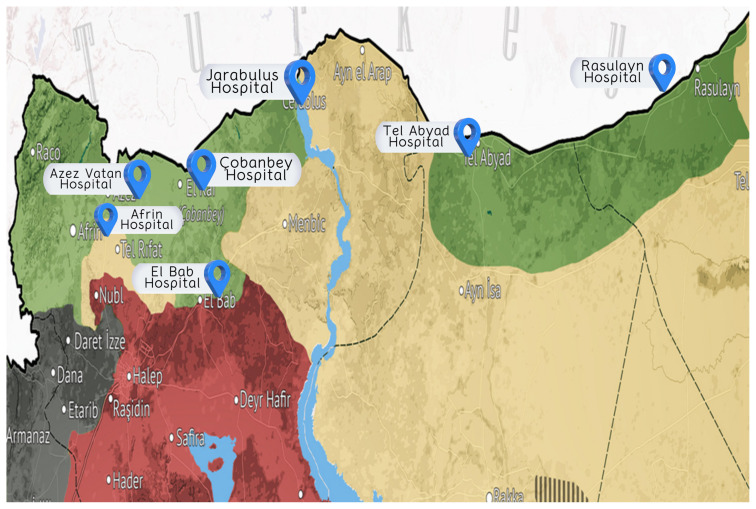
Localizations of hospitals in northern Syria included in the study.^1^ ^1^Mepanews (2020). Suriye’de son durum haritası (online).Website https://www.mepanews.com/suriye-son-durum-haritasi-subat-2020-33767h.htm [accessed 20 May 2022].

In northern Syria, there are hospitals that were opened by Turkey under the framework of humanitarian aid. They often provide consultation services. Syrian doctors, nurses, and other medical staff work in these hospitals and provide care to people in northern Syria. This study was conducted in the hospitals in Afrin, Azez Vatan, Jarablus, Telabyad, Rasulayn, El Bab, and Çobanbey (Figure 1).

### 2.3. Patient selection

**Figure 2:**
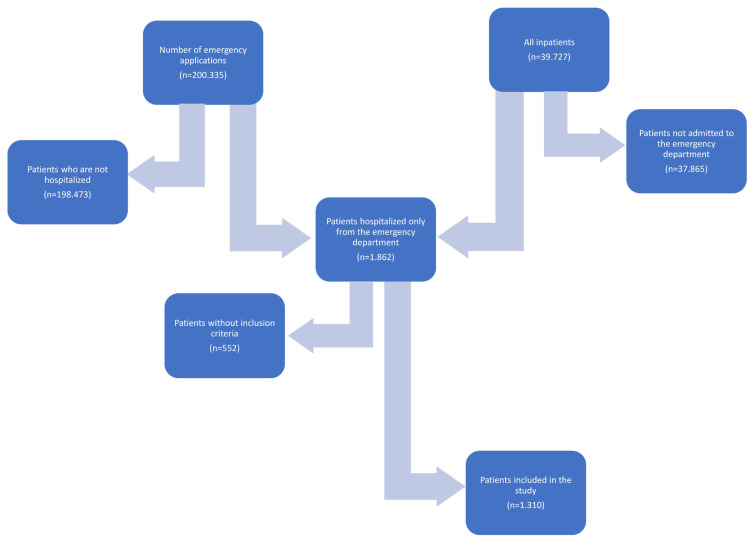
Flow chart of patients included and excluded from the study

Patients over the age of 18 who were hospitalized in the emergency service in these hospitals were included in the study. Patients who were discharged from the emergency room, brought to the emergency room in a cardiopulmonary arrest state, died in the emergency room, were pregnant, were transferred from the emergency room to a place not included in the study, or whose clinical data could not be obtained were excluded from the study (Figure 2).

### 2.4. Obtaining data

Demographic data from hospitalized patients, complaints on admission, department/date/time of hospitalization, presence of trauma, ability to stand on their own, vital signs, oxygen support status, presence of hypercapnia, consciousness status, Glasgow Coma Scale (GCS) value, and presence of abnormal electrocardiography (ECG) findings were recorded using forms. Left and right bundle branch block, pathological Q waves, second and third-degree AV blocks, atrial fibrillation, ventricular fibrillation or tachycardia, left and right axis deviation, left and right ventricular hypertrophy, ST-segment and/or T-wave abnormalities, and a QTc interval >450 ms were accepted as abnormal ECG findings.

Patients who were hospitalized in the emergency department were followed up by the local healthcare workers from the relevant clinic. During the period of the research, follow-up records were made manually as there was no digital archive file system in the hospitals included in the study. Patients’ data were entered into the form papers prepared for the study by the local healthcare workers. In line with these records, the patients’ National Early Warning Score 2 (NEWS2)^2^ (Table 1), Rapid Acute Physiology Score (RAPS) (Table 2) [13], Rapid Emergency Medicine Score (REMS) (Table 2) [10] and HOTEL Score (hypotension, oxygen saturation, low temperature, electrocardiogram, loss of independence) (Table 3) [19] were calculated.

For the evaluation of in-hospital mortality, hospitalized patients were studied over 28 days in line with the literature [4,19]. Patients who were discharged and those who were alive after 28 days were included in the survivor group. Patients who were hospitalized in the ICU for one day or more during their hospital stay were considered as hospitalized in the ICU. According to the data, the patients were split into two separate groups as survivors and patients who died, and those who were hospitalized in the wards and those in the ICUs. Acceptance parameters and scores were analyzed using statistical methods by comparing groups.

### 2.5. Statistics

Statistical analyses for the study were performed using the Statistical Package for Social Sciences version 25.0 software for Windows (IBM SPSS Statistics for Windows, version 25.0, IBM Corporation, Armonk, NY, USA). Normality assumptions for the quantitative variables were made using Kolmogorov-Smirnov and Shapiro-Wilk tests. The descriptive statistics of the variables were summarized as mean ± standard deviation, median (minimum-maximum), and n (%). The binary comparison of continuous variables was done with Mann-Whitney U test or student t test according to data distribution normality. The comparison of categorical variables between groups was done using the chi-square test or Fisher Freeman exact test. Univariate logistic regression analysis was performed to determine the risk values of four different scoring methods in estimating mortality and hospitalization events. ROC curve analysis was used to determine whether the scoring methods had a cut-off value for mortality and hospitalization in the ICU. In all statistical analysis, cases with a “p” value less than 0.05 were interpreted as statistically significant.

## 3. Results

A total of 1310 patients who had been hospitalized for an emergency were included in the study. Of these patients, 374 (28.5%) were hospitalized in ICUs and 936 (71.5%) in wards. The mean age of the patients was 48.2 ± 21.0 years. The mean age of patients treated in the ICU was higher than that of patients in the wards. The mean age of patients who were alive was lower than that of patients who died (p < 0.05). The proportion of older patients (>65 years) hospitalized in the ward compared with those hospitalized in the ICU, and the proportion of patients who survived, was lower than that of patients who died (Table 4) (p < 0.05).

When evaluating the distribution of patients’ hospitalization times during the day, most hospitalizations occurred during working hours (08:00–16:00). The rate of admissions to the wards during working hours was higher. Outside working hours (16:00–08:00), the rate of patients admitted to the ICU was higher (p < 0.05). When examining the relationship between survival status and hospitalization hours, the ratio of the number of hospitalizations during working hours to the number of hospitalizations during the whole day was higher in surviving patients than in deceased patients. However, the ratio of the number of hospitalizations outside working hours to the number of hospitalizations during the whole day was higher in deceased patients.

When examining the relationship between survival status and hospitalization hours, the rate of patients hospitalized during working hours was higher than that of patients who died. Compared with the hours of hospitalization outside working hours, the rate of patients who died was higher than that of those who survived (p < 0.05) (Table 4).

While most patients admitted to the ICU were from the ED, most patients admitted to the ward were patients referred from other hospitals (p < 0.05). While more than half of the patients who survived were referred to other hospitals, most patients who died were admitted from the ED (p < 0.05) (Table 4). When 668 patients who were referred from other hospitals were evaluated in more detail, it was found that 1.6% of these patients (n = 11) died and 12.4% of these patients (n = 83) were admitted to the ICU.

Trauma had occurred in 7.6% of the patients. However, 13.8% of patients who died had trauma, whereas this rate decreased to 7.2% in patients who survived (p < 0.05) (Table 4).

When evaluating the vital signs of the patients included in the study, the mean values of systolic, diastolic, and mean blood pressure were higher in the patients admitted to the ICU than in the hospitalized patients and were higher in the surviving patients than in the deceased patients (p < 0.05). The deceased patients had a higher mean heart rate than the surviving patients and that the ICU admitted patients had a higher mean heart rate than the hospitalized patients (p < 0.05) (Table 4).

When respiratory parameters were evaluated, the mean saturation values of patients admitted to the ICU were lower than those of patients admitted to the ward and those of deceased patients compared with living patients. The rate of oxygen support received was higher in patients hospitalized in the ICU than in patients hospitalized in the ward and in deceased patients compared with living patients (p < 0.05) (Table 4).

Patients in the ICU had a higher rate of abnormal ECG findings than patients hospitalized in the ward and deceased patients compared with surviving patients (p < 0.05) (Table 4).

When patients were assessed from a cognitive standpoint, the rate of clarity of consciousness was higher in patients in the ward than in patients in the ICU. The mean GCS of patients in the ward were higher than those of patients in the ICU and those of patients who survived were higher than those of patients who died. Additionally, some patients in the ward could stand independently, whereas none of the patients who died could stand independently (p < 0.05) (Table 4).

When the distribution of patients by prognosis scores was evaluated, it was found that the median values of HOTELS, RAPS, REMS, and NEWS2 were higher in ICU patients than in ward patients and were higher in patients who died than in surviving patients (p < 0.05) (Table 4).

Univariate logistic regression analysis was performed using 4 different scoring systems to assess the risk of death and hospitalization in the ICU (Table 5). When the results of these analysis were evaluated in terms of mortality risk, one unit increase of HOTELS, RAPS, REMS, and NEWS2 scoring increased the mortality risk by 5.1 times, 2.3 times, 1.6 times and 1.6 times, respectively. Additionally, when the results of these analyses are considered in terms of the risk of ICU hospitalization, one unit increase of HOTELS, RAPS, REMS, and NEWS2 scoring increased the mortality risk by 4.2 times, 2.0 times, 1.5 times, and 1.5 times, respectively. Notably, the results of the analysis of REMS and NEWS2 are identical regarding the risk of ICU hospitalization.

**Figure 3:**
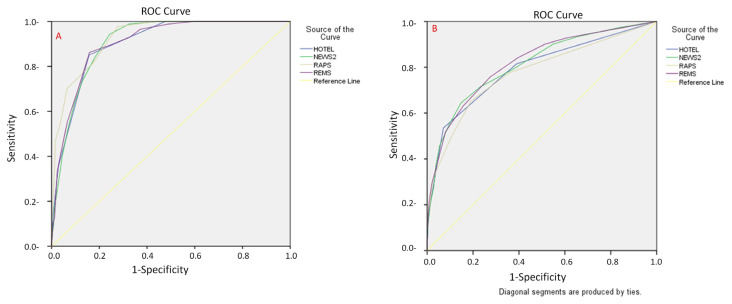
ROC curve of 4 prognostic scoring systems with respect to patient groups. A: the deceased patient group; B: for the patients hospitalized in the ICU group.

ROC analysis was performed to determine whether scoring systems have a cut-off value for detecting in-hospital mortality (Table 6). Due to the ROC analysis, the four different scoring systems used had value in detecting in-hospital mortality (p < 0.05). REMS was shown to have the highest sensitivity and specificity amongst the four systems, at 86.2% and 84.1%, respectively. With a cut-off value of 6.5, the negative and positive predictive value of REMS was 98.84% and 27.88%, respectively. The detailed statistical results are shown in Table 6. It can be seen that the area under the ROC curves in Figure 3A is statistically significant (p < 0.05).

When the areas under the ROC curve were examined, it was found that all four scoring systems were effective in predicting mortality. The statistical significance of the RAPS, which had the largest AUC value, was slightly stronger than that of the other scores. It was found that the ROC curves of the REMS and HOTELS were very similar (Figure 3A).

ROC analysis was performed to determine whether the scoring systems had a cut-off value for detecting the risk of hospitalization in the ICU (Table 6). According to these results, the area under the ROC curve of the 4 different scoring methods was statistically significant (p < 0.05). Examining the ROC curves, each scoring system did not make a sharp break after moving away from the direct diagonal (Figure 3B). For these results, the sensitivity values of the cut-off values given by the scoring systems remained below 0.70 and RAPS had the highest sensitivity (65.2%) amongst the four systems with a cut-off value of 1.5. The negative and positive predictive values of RAPS were found to be 85.34% and 57.68%, respectively. The ROC curves of the 4 critical care scoring systems studied were similar. This indicates that none of the scoring systems are overtly superior.

## 4. Discussion

In this study, scoring systems were evaluated for prediction of mortality and intensive care admission risk in patients hospitalized from the emergency department. The study identified significant findings among the scoring systems. For mortality, REMS was shown to have the highest sensitivity and specificity at 86.2% and 84.1%, respectively (NPV 98.84%, PPV 27.88%). For ICU hospitalization, RAPS was shown to have the highest sensitivity at 65.2% (NPV 85.34%, PPV 57.68%).

Wars and conflicts across the world greatly affect civilians biopsychosocially in the regions where these events occur [20,21]. Internal and external migration is increasing and problems in accessing vital basic needs create humanitarian crises [22]. International governmental and private aid organizations are providing humanitarian assistance in these regions. In this context, the provision of health services is a basic need.^3^ Although emergency medical care is the primary focus in conflict areas, public health needs, advanced emergencies, and inpatient care should are also needed in the period leading up to the comprehensive postconflict reconstruction [23]. The need for emergency and inpatient treatment services to address endemic and pandemic health problems in postconflict regions is also crucial.

Northern Syria is home to 4 million people who have been victims of retaliation and have suffered terrorism.^4^,^5^ Now that terrorism has been largely removed from the region, there is a need for comprehensive emergency medical care and hospital services.^6^,^7^ These services are necessary to ensure that people in the affected region have access to treatment. Furthermore, they can help to ensure that this region does not become a new pandemic hotspot.

In northern Syria, internal turmoil has left the health system dysfunctional, the number of medical professionals has declined, and health facilities have been severely damaged ordestroyed.^8^,^9^,^10^

To date, many nongovernmental organizations have attempted to provide fragmented healthcare services to local health workers using humanitarian aid funds.^11^,^12^

Since 2016, safe areas have been created by removing the threat of terrorism in northern Syria. Alongside restoring old and damaged hospitals in the region, Turkey has built new hospitals in these regions as part of their humanitarian aid efforts^13^ [24]. This study evaluated patients admitted from the emergency departments in hospitals that were opened as part of humanitarian aid efforts in northern Syria, or those admitted and hospitalized in an external center for emergency reasons.

Trauma patients accounted for 7.6% of the patients included in the study. The incidence of trauma in patients requiring hospitalization for emergencies was expected to be higher in places of internal unrest, such as northern Syria. However, in this postwar troubled region, hospitals where patients can stay for internal illness may be limited. However, a COVID-19 outbreak at the time of the study may help to explain the low trauma rate among general emergency hospitalizations.

In the literature, older age has been shown to be a negative predictive factor. Age is an easily obtained piece of patient information and is a parameter used in the calculations of prognostic scoring systems developed for some diseases or conditions [25,26]. In this study, parallel to the literature, the mean ages of patients hospitalized in the ICU were higher than those of patients hospitalized in the wards, and those of deceased patients were higher than those of living patients.

Referrals may be necessary in rural areas of middle- and low-income countries because of the lack of qualified healthcare workers [27]. In a study conducted in Thailand, it was found that there was no standardized procedure for transferring patients. Furthermore, 9% of patients who were referred from other hospitals and required immediate hospitalization died [25]. This study found that 1.6% of 668 patients (n = 11) who came to the hospital with a referral died (n = 83) and 12.4% were treated in the ICU. Differences in demographic characteristics and diagnostic rates may lead to differences in mortality rates among referred patients. However, this study also showed that the mortality rate of patients hospitalized from the ED was higher than that of referred patients. Although patients admitted by referral were urgent cases, it was necessary to be more selective in admitting referral patients due to limited resources because of patient density. This may help to explain the lower mortality rate among patients who were referred.

In a study conducted in Spain, in which different scales were compared in the prediction of mortality, it was found that the deceased patient group had lower average blood pressures and O_2_ saturation values and higher average heart rates compared to the surviving patient group [28]. In a study on scoring systems in Turkey, it was shown that patients hospitalized in the ICU had lower blood pressure and saturation values and higher pulse rates than those hospitalized in the ward [4]. These vital signs used in the creation of prognostic scoring systems were similar to those in the literature when comparing the deceased-surviving and ICU-ward inpatient groups in this study. Vital signs also retain their own prognostic significance and their combinations are used in scoring systems to achieve stronger predictive models. In some countries, multicenter studies evaluate these validities at the national level [29].

A normal ECG has been shown to be a strong predictor of survival. It has been calculated that mortality rate raises by 9% when combined with other parameters used in mortality indices [26]. In some studies of geriatric patients, ECG abnormalities are common in deceased patients [4]. In this study, in accordance with the literature, it was observed that there were more patients with abnormal ECGs in the deceased and ICU groups than in the living and hospitalized groups. However, if cardiac pathology is not considered in the emergency department, it may seem unnecessary to obtain ECGs in patients. However, the presence of an abnormal ECG may support a negative prognosis even if the patient does not have cardiac disease in cases requiring urgent hospitalization.

In a study to predict prognosis in patients hospitalized in the emergency departments in China, GCS alone was found to be a predictive factor for hospitalization and mortality [30]. In another study conducted in Spain, the GCS was found to be lower in the deceased patient groups (median: 11) [28]. In the study by Başpınar et al., it was shown that the rate of unconsciousness was higher in patients hospitalized in the ICU compared to patients hospitalized in the ward and the mean GCS was 13.5 [4].

In this study, the GCS value was lower in patients admitted to the ICUs, with an average of 12.2, compared to those admitted to the ward. In the group of patients who died, the mean GCS was 7.1, which was lower than that of the patients who survived. The rate of loss of consciousness was found to be higher in the groups of deceased and ICU patients than in the groups of surviving and ward patients. Although similar results have not been shown in other studies, this indicates that GCS and cognitive impairment have negative predictive significance for patients because of parallel outcomes.

HOTELS is a scoring system that estimates mortality risk based on 5 readily available variables. It can be calculated quickly and easily without incurring additional costs for medical personnel [26]. In the study in which the HOTEL scale was first defined (AUC value 0.8651), it was shown to have a strong mortality predictive ability. Recent studies have also supported its usefulness in predicting patient mortality [26]. In this study, in parallel with the literature, HOTELS was found to be statistically significant in both mortality and ICU hospitalization predictions.

Statistical analysis in the literature suggests that the Taiwanese validation study of the HOTELS developed for mortality estimation found an AUC value of 0.931 (95% CI, 0.901–0.962) and found that mortality increased by 5.56 times for each unit increase. In other studies, the cut-off value for HOTELS was set at 2 and HOTELS proved to be useful for estimating mortality [19,31]. In this study, the cut-off value was 1.5 with an AUC of 0.903, sensitivity of 85.1%, and specificity of 84.3%, although it was not similar.

The literature further suggests that although there are individual validation studies for HOTELS in different patient groups, these studies are mostly based on comparing scoring systems. In a study comparing HOTELS, REMS and “REMS without age” for ICU hospitalizations in geriatric patients, the AUC was found to be 0.827, 0.772, and 0.760, respectively, with the performance of HOTELS proving to be better [19,32].

A study by Nakhjavan-Shahraki et al. included more than two thousand trauma patients. The optimal cut-off value for the risk of mortality and poor outcome was 3 at REMS and 2 at RAPS. The study found that the REMS model predicted mortality and poor outcome in trauma patients better than the RAPS model. However, both models had acceptable predictive values for trauma patients [32]. In another study on trauma patients, it was shown that the mortality rate of trauma patients increased significantly with the increase in REMS [33]. Indeed, it has been determined that results similar to trauma scoring systems are obtained in some traumas for REMS [11].

For other patient subgroups, it was determined that REMS had an acceptable predictive value for mortality in some infectious diseases [34].

In a study comparing scoring systems for evaluating acute neurological conditions in the ED, RAPS was found to be one of the scoring systems with high predictive power for mortality prediction in ICU patients [35].

In studies examining general patient populations hospitalized in the ED, REMS was superior to RAPS for mortality. In these samples, the REMS model proved to be a powerful tool for predicting mortality in nonsurgical patients [11,12].

In a study conducted in China by Wei et al., REMS was shown to have strong predictive value for mortality, length of stay, hospital stay, and outcomes in adult patients in the ED [30].

In a study by Bulut et al. that evaluated more than two thousand patients, the REMS cut-off median score was 6.5 in the patient groups that died and 6 in the patients treated in the ward and ICU [36].

In this study, sensitivity was 86.2% and specificity was 84.1% when the REMS cut-off value for mortality was 6.5, whereas sensitivity was 81.6% and specificity 82.5% when the RAPS cut-off value was 2.5. Furthermore, sensitivity was 63.1% and specificity was 84.3% when the REMS cut-off value was 5.5. When the RAPS cut-off value was 1.5, sensitivity was 65.2% and specificity was 80.9%. The results of this study align with studies with strong results in the literature in estimating mortality for REMS and RAPS. The REMS value was stronger in estimating admissions than RAPS, in agreement with the literature.

A publication comparing NEWS with 33 different scoring systems found that no scoring system was more powerful in predicting death and critical care than NEWS [29]. In late 2017, Hodgson et al. published the NEWS2 scale, which is considered a better alternative to NEWS and uses hypercapnia instead of SpO2 [37].

Considering the detailed statistical analysis in the study by Rodriguez et al. when evaluating all patients admitted to the ED, the NEWS2 cut-off value of 9 was found to predict mortality with a sensitivity of 79.9% and specificity of 84.5%. Evaluation of the subgroups in this sample also showed that the cut-off value was 7 for medical conditions and 10 for trauma [28].

In this study, the cut-off value for the risk of death in NEWS2 was 6.5, sensitivity was 83.9%, and specificity was 81.9%. Regarding the risk of ICU hospitalization, the sensitivity was 64.4% and the specificity was 85.3% with a cut-off value of 5.5. Although the publications in the literature and sample in this study include disparate patient groups and the values for sensitivity and specificity are different, this study’s cut-off values are largely in agreement with those in the literature.

RAPS makes its predictions based on 4 parameters: heart rate, blood pressure, respiratory rate, and GCS findings. Only a sphygmomanometer is required to calculate the RAP score. REMS was developed from RAPS, and two more parameters were added to those used in RAPS to achieve more effective prediction [13,30]. HOTELS and NEWS2 also use 6 parameters^2^ [14]. In the REMS model, the age parameter was added to RAPS. Although there is no cost in obtaining age data, there may be problems in determining this data because the birth years of individuals are not accurately recorded or for patients with communication problems [19]. Regarding equipment requirements, unlike RAPS, a thermometer is required for the calculation of the NEWS2 and HOTELS models^2^ [14]. In addition, three models require a saturation device for calculation, which is more expensive than RAPS. The biggest challenge is posed by HOTELS, which requires an additional ECG device [26]. In developed countries, attempts are being made to make more accurate estimates with more complex models that use more parameters. ECGs, thermometers, and saturometers are easily available in developed countries [38]. However, these devices are not found in underdeveloped healthcare facilities where there are physical and financial challenges or the devices are broken and old. However, accurate estimates with the lowest parameters in terms of cost-effective and efficient use of time with limited resources can bring more benefits, especially in underdeveloped regions and countries [5]. In this study, the statistical power of RAPS was slightly stronger than that of the other scores. It would be beneficial to use the RAPS model due to its high predictive value and fewer parameters in emergency medical care in similar regions.

Diagnostic subgroups were not evaluated in this study. Syrian health workers make decisions about the treatment and hospitalization of the patients included in the study. In this context, diagnosis, treatment, hospitalization, and referral protocols are closely linked but may not be fully standardized. They could not be standardized because physicians had different perspectives such as ECG abnormality classification, low saturation causes, and unconsciousness reasons. Physicians were not included in the evaluation. Although the impact of this is the limitation of comparisons between deceased and surviving patient groups, it may be beneficial for comparisons between patient groups treated in the ICU and on the wards.

In conclusion, while all four scoring systems were effective in predicting mortality, the statistical significance of the RAPS was slightly stronger and REMS was shown to have the highest sensitivity and specificity of the four systems. Furthermore, RAPS was shown to have the highest sensitivity predicting risk of hospitalization in the ICU of the four systems.

As in developed countries, the use of scoring systems in regions where healthcare systems have deteriorated or are being revitalized, and where there are limited health facilities and personnel, will contribute to cost-effective healthcare service delivery. This study, which includes a perspective from northern Syria, will lead to future studies in similar regions.

## Figures and Tables

**Table 1:** The National Early Warning Score 2 scale.

Physiological parameter	Score						
	3	2	1	0	1	2	3
**Respiration rate (per minute)**	≤8		9–11	12–20		21–24	≥25
**SpO** **_2_** **(%)**	≤91	92–93	94–95	≥96			
**Air or oxygen?**		Oxygen		Air			
**Systolic blood pressure (mmHg)**	≤90	91–100	101–110	111–219			≥220
**Pulse (per minute)**	≤40		41–50	51–90	91–110	111–130	≥131
**Consciousness**				Alert			CVPU
**Temperature (°C)**	≤35.0		35.1–36.0	36.1–38.0	38.1–39.0	≥39.1	

**Table 2:** Rapid Acute Physiology Score (RAPS) and Rapid Emergency Medicine Score (REMS) scale.

RAPS variable	RAP score				

	0	+1	+2	+3	+4

**PR (/min)**	70–109		55–69	40–54	≤39
			110–139	140–179	≥180

**MAP (mmHg)**	70–109		50–69	130–159	≤49
			110–129		≥160

**RR (/min)**	12–24	10–11	6–9	35–49	≤5
		25–34			≥50

**GCS**	≥14	11–13	8–10	5–7	4

REMS variable	REM score						

	0	+1	+2	+3	+4	+5	+6

**Age (years)**	<45		45–54	55–64		65–74	>74

**PR (/min)**	70–109		55–69	40–54	≤39		
			110–139	140–179	>179		

**MAP (mmHg)**	70–109		50–69	130–159	≤49		
			110–129		>159		

**RR (/min)**	12–24	10–11	6–9	35–49	≤5		
		25–34			>49		
**GCS**	14 or 15	11–13	8–10	5–7	3 or 4		

**SpO** **_2_** ** (%)**	>89	86–89		75–85	<75		

PR: pulse rate; MAP: mean arterial pressure; RR: respiratory rate; GCS: Glasgow Coma Scale; SpO2: peripheral oxygen saturation.

**Table 3:** HOTEL (hypotension, oxygen saturation, temperature, electrocardiogram, loss of independence) scoring system.

Variables	Score
Systolic blood pressure <100 mmHg	1
Oxygen saturation <90%	1
Temperature <35.0 °C	1
Abnormal electrocardiogram	1
Loss of independence	1

**Table 4:** Characteristics of the study patients according to the surviving and deceased status and the patients’ groups regarding the hospitalization to the intensive care unit and to the ward.

Variables		Total N = 1310	Surviving N = 1223	Deceased N = 87	p	Hospitalization		
**Hospitalization departmen**t	**ICU**	374 (28.5%)	290 (23.7%)	84 (96.6%)	0.000^b^	**ICU** N = 374	**Ward** N = 936	**p**
	**Another ward**	936 (71.5%)	933 (76.3%)	3 (3.4%)				
**Overall age average**		48.23 ± 21.01	47.16 ± 20.69	63.37 ± 19.71	0.000^a^	60.69 ± 18.89	43.26 ± 19.72	0.000^1^
**18–65 years average**		39.44 ± 15.00	38.95 ± 14.89	49.44 ± 13.86	0.000^a^	38.95 ± 14.89	49.44 ± 13.86	0.000^1^
**Average over 65 years old**		77.08 ± 8.08	76.68 ± 7.48	79.75 ± 11.02	0.025^a^	76.68 ± 7.48	79.75 ± 11.02	0.084^1^
**18–65 years n (%)**		1004 (76.6%)	957 (78.3%)	47 (54.0%)	0.000^b^	957 (78.3%)	47 (54.0%)	0.000^2^
**Over 65 years n (%)**		306 (23.4%)	266 (21.7%)	40 (46.0%)		266 (21.7%)	40 (46.0%)	
**Sex**	**Male**	642 (49.0%)	597 (48.8%)	45 (51.7%)	0.600^b^	194 (51.9%)	448 (47.9%)	0.190^2^
	**Female**	668 (51.0%)	626 (51.2%)	42 (48.3%)		180 (48.1%)	488 (52.1%)	
**Hospitalization time**	**08:00–16:00**	980 (74.8%)	929 (76.0%)	51 (58.6%)	0.000^b^	225 (60.2%)	755 (80.7%)	0.000^2^
	**16:00–00:00**	236 (18.0%)	206 (16.8%)	30 (34.5%)		114 (30.5%)	122 (13.0%)	
	**00:00–08:00**	94 (7.2%)	88 (7.2%)	6 (6.9%)		35 (9.4%)	59 (6.3%)	
**Hospitalization from the emergency service n (%)**		642 (49.0%)	566 (46.3%)	76 (87.4%)	0.000^b^	291 (77.8%)	351 (37.5%)	0.000^2^
**Hospitalization with referral n (%)**		668 (51.0%)	657 (53.7%)	11 (12.6%)		83 (22.2%)	585 (62.5%)	
**Trauma**	**Yes n (%)**	100 (7.6%)	88 (7.2%)	12 (13.8%)	0.042^d^	35 (9.4%)	65 (6.9%)	0.137^2^
	**No n (%)**	1210 (92.4%)	1135 (92.8%)	75 (86.2%)		339 (90.6%)	871 (93.1%)	
**Ability to stand on their own**	**Yes n (%)**	1138 (86.9%)	1138 (93.0%)	0 (0.0%)	0.000^b^	233 (62.3%)	905 (96.7%)	0.000^2^
	**No n (%)**	172 (13.1%)	85 (7.0%)	87 (100.0%)		141 (37.7%)	31 (3.3%)	
**Systolic blood pressure**		123.26 ± 21.70	123.63 ± 20.54	118.02 ± 33.80	0.020^a^	128.49 ± 30.43	121.17 ± 16.56	0.020^1^
**Diastolic blood pressure**		73.47 ± 12.79	73.80 ± 12.22	68.80 ± 18.63	0.000^a^	74.99 ± 17.17	72.86 ± 10.50	0.007^1^
**Arterial pressure**		89.98 ± 14.47	90.32 ± 13.70	85.10 ± 22.26	0.001^a^	92.70 ± 20.40	88.89 ± 11.09	0.001^1^
**Fever**		36.54 ± 0.55	36.54 ± 0.54	36.62 ± 0.66	0.175^a^	36.64 ± 0.63	36.50 ± 0.51	0.175^1^
**Pulse**		87.73 ± 15.64	87.10 ± 14.44	96.57 ± 26.03	0.000^a^	91.92 ± 22.10	86.06 ± 11.75	0.000^1^
**Respiratory rate**		19.87 ± 6.29	20.27 ± 6.13	14.26 ± 5.87	0.000^a^	19.80 ± 7.94	19.89 ± 5.51	0.813^1^
**O2 saturation**		95.58 ± 5.45	95.77 ± 5.42	92.96 ± 5.10	0.000^a^	92.98 ± 6.96	96.63 ± 4.29	0.000^1^
**Does the patient receive O2 support?**	**Yes n (%)**	376 (28.7%)	292 (23.9%)	84 (96.6%)	0.000^b^	263 (70.3%)	113 (12.1%)	0.000^2^
	**No n (%)**	934 (71.3%)	931 (76.1%)	3(3.4%)		111 (29.7%)	823 (87.9%)	
**Presence of hypercapnia**	**Yes n (%)**	92 (7.0%)	74 (6.1%)	18 (20.7%)	0.000^d^	80 (21.4%)	12 (1.3%)	0.000^2^
	**No n (%)**	1218 (93.0%)	1149 (93.9%)	69 (79.3%)		294 (78.6%)	924 (98.7%)	
**Consciousness status**	**Conscious N (%)**	827 (63.1%)	827 (67.6%)	0 (0.0%)	0.000^b^	141 (37.7%)	686 (73.3%)	0.000^2^
	**Unconscious n (%)**	483 (36.9%)	396 (32.4%)	87 (100.0%)		233 (62.3%)	250 (26.7%)	
**Glasgow Coma Scale**			14.29 ± 1.19	7.09 ± 2.77	0.000^a^	12.19 ± 3.37	14.46 ± 1.02	0.000^1^
**Presence of abnormal ECG findings**	**Yes n (%)**	232 (17.7%)	173 (14.1%)	59 (67.8%)	0.000^b^	184 (49.2%)	48 (5.1%)	0.000^2^
	**No n (%)**	1078 (82.3%)	1050 (85.9%)	28 (32.2%)		190 (50.8%)	888 (94.9)	
**Median of scores**	**HOTELS**	1 (0–4)	0 (0–4)	2 (1–4)	0.000^e^	2 (0–4)	0 (0–4)	0.000^3^
	**RAPS**	0 (0–13)	0 (0–9)	5 (1–13)	0.000^e^	2 (0–13)	0 (0–8)	0.000^3^
	**REMS**	3 (0–20)	2 (0–16)	9 (2–20)	0.000^e^	7 (0–20)	2 (0–13)	0.000^3^
	**NEWS2**	3 (0–16)	3 (0–16)	10 (4–16)	0.000^e^	7 (0–16)	3 (0–14)	0.000^3^

1 independent t-test;

2 chi-square test;

3 Mann-Whitney U test;

a independent t-test;

b chi-square test;

c Fisher Freeman exact test;

d continuity correction;

e Mann-Whitney U test

**Table 5:** Results of univariate logistic regression analysis for the risk of patient death and hospitalization in the intensive care unit regarding to 4 prognostic scoring systems.

Mortality scores	B	SE	Wald	p	Exp (B)	95% CI for Exp (B)	
						Lower	Upper
*HOTELS*	1.633	0.138	140.12	0.000	5.117	3.905	6.705
*RAPS*	0.820	0.066	156.51	0.000	2.271	1.997	2.582
*REMS*	0.443	0.039	131.391	0.000	1.557	1.443	1.679
*NEWS 2*	0.438	0.037	139.27	0.000	1.550	1.441	1.667
**ICU scores**							
*HOTELS*	1.448	0.090	258.79	0.000	4.255	3.567	5.076
*RAPS*	0.678	0.045	223.814	0.000	1.971	1.803	2.154
*REMS*	0.395	0.024	271.689	0.000	1.484	1.416	1.555
*NEWS 2*	0.395	0.024	262.257	0.000	1.484	1.415	1.556

**Table 6:** ROC analysis results risk of 4 prognostic scoring systems for in-hospital mortality and hospitalization in the ICU.

Scores		AUC±SE	95%CI	p	Cut-off value	Sensitivity	Specificity	PPV	NPV
**Mortality**	*HOTEL*	0.903 ± 0.013	0.878–0.928	0.000	1.5	0.851	0.843	27.81	98.75
	*RAPS*	0.928 ± 0.011	0.907–0.949	0.000	2.5	0.816	0.825	21.63	99.46
	*REMS*	0.906 ± 0.013	0.881–0.931	0.000	6.5	0.862	0.841	27.88	98.84
	*NEWS 2*	0.909 ± 0.011	0.889–0.930	0.000	6.5	0.839	0.819	23.22	98.62
**ICU**	*HOTEL*	0.792 ± 0.015	0.763–0.821	0.000	1.5	0.535	0.929	75.18	83.33
	*RAPS*	0.776 ± 0.015	0.746–0.807	0.000	1.5	0.652	0.809	57.68	85.34
	*REMS*	0.818 ± 0.013	0.792–0.844	0.000	5.5	0.631	0.843	61.61	85.11
	*NEWS 2*	0.811 ± 0.014	0.784–0.838	0.000	5.5	0.644	0.853	63.58	85.71

## Footnotes

1 Institute for Economics & Peace (2022). Global Terrorism Index 2022: Measuring the Impact of Terrorism [online]. Website http://visionofhumanity.org/resources [accessed 22 June 2022].

2 Royal College of Physicians (2017).National Early Warning Score 2 Standardising the assessment of acute-illness severity in the NHS [online]. Website https://www.rcplondon.ac.uk/projects/outputs/national-early-warning-score-news-2 [accessed 17 June 2022].

3 World Health Organization (2022). National workforce capacity to implement the essential public health functions including a focus on emergency preparedness and response: roadmap for aligning WHO and partner contributions [online]. Website: https://apps.who.int/iris/handle/10665/354384 [accessed 20 June 2022].

4 Dailysabah (2022). Turkey to build 200000 homes for 1M Syrians to resettle voluntarily: Erdoğan [online].Website https://www.dailysabah.com/politics/turkey-to-build-200000-homes-for-1m-syrians-to-resettle-voluntarily-erdogan/news [accessed 17 June 2022].

5 Europian Unions (2022). European Civil Protection and Humanitarian Aid Operations[online]. Website https://civil-protection-humanitarian-aid.ec.europa.eu/where/middle-east/syria_en [accessed 17 June 2022].

6 The United Nations Refugee Agency (2022). Syria emergency [online]. Website https://www.unhcr.org/syria-emergency.html [accessed 20 June 2022].

7 Union of Medical Care and Relief Organizations (2016). Syrian crisis [online]. Website https://www.uossm.org/reports?gclid=Cj0KCQjwzLCVBhD3ARIsAPKYTcRN4UJQTjXJjqcaS9D5Lc9U1A_LURq1qZ8qxGlJwOISvzc4sPgV1AaArZ5EALw_wcB [accessed 20 June 2022].

8 United Nations Office for the Coordination of Humanitarian Affairs (2021). Destruction, Obstruction, and Inaction: The Makings of a Health Crisis in Northern Syria [online]. Website https://reliefweb.int/report/syrian-arab-republic/destruction-obstruction-and-inaction-makings-health-crisis-northern [accessed 17 June 2022].

9 United Nations Office for the Coordination of Humanitarian Affairs (2022). A Heavy Price to Pay: Attacks on Healthcare Systems in Syria 2015–2021 [online]. Website https://reliefweb.int/report/syrian-arab-republic/heavy-price-pay-attacks-healthcare-systems-syria-2015-2021-may-2022 [accessed 17 June 2022].

10 World Health Organization (2018). Whole of Syria Health Cluster Bulletin [online]. Website https://www.humanitarianresponse.info/sites/www.humanitarianresponse.info/files/2018/11/Whole%20of%20Syria%20Health%20Cluster%20Bulletin%20October%202018_final.pdf [accessed 17 June 2022].

11 UNICEF (2022). Whole of Syria Humanitarian Situation Report for January – December 2021 [online]. Website https://reliefweb.int/report/syrian-arab-republic/unicef-whole-syria-humanitarian-situation-report-january-december-2021 [accessed 20 June 2022].

12 United Nations Office for the Coordination of Humanitarian Affairs (2022). Snapshot of Whole of Syria health sector response (4Ws) [online]. Website https://www.humanitarianresponse.info/sites/www.humanitarianresponse.info/files/documents/files/wos_4ws_apr_2022_final.pdf [accessed 20 June 2022].

13 T.C. İletişim Başkanlığı (2019). Sınır ötesine sağlık yardımı [online]. Website https://www.iletisim.gov.tr/turkce/yerel_basin/detay/sinir-otesine-sagik-yardimi [accessed 20 June 2022].
